# Controlled expansion and differentiation of mesenchymal stem cells in a microcarrier based stirred bioreactor

**DOI:** 10.1186/1753-6561-5-S8-P55

**Published:** 2011-11-22

**Authors:** Sébastien Sart, Abdelmounaim Errachid, Yves-Jacques Schneider, Spiros N Agathos

**Affiliations:** 1 Université Catholique de Louvain / Institut des Sciences de La Vie, Belgium; 2 Laboratory of Bioengineering (GEBI), Place Croix du Sud, 2/19, 1348 Louvain-la-Neuve, Belgium; 3 Laboratory of Cellular Biochemistry, Place Croix du Sud, 4/5 box 3, 1348 Louvain-la-Neuve, Belgium

## Introduction

Cell based therapy requires great numbers of cells in a functional state permitting their *in vivo* implantation for the restoration of tissue homeostasis. Three main parameters are believed to be essential for such a purpose: an appropriate cell population, a suitable scaffold and appropriate physical / biochemical factors enabling proper expansion and *in vitro* cell differentiation. In recent years, mesenchymal stem cells (MSCs) have been attracting a lot of interest in this field, because of their differentiation potential and their trophic factor secretion abilities. The aim of this work is to perform a rational analysis of key factors involved in the efficient proliferation and differentiation of MSCs, in the context of a stirred microcarrier (MC)-based bioreactor.

## Materials and methods

MSCs from external ear (E-MSCs) and bone marrow stroma (BM-MSCs) were extracted from *Wistar* rats, selected and cultivated on plastic dishes as previously described [1]. The differentiation potential of E-MSCs along the adipogenic, osteogenic and chondrogenic pathways was established and assessed by staining (respectively Oil red O, von Kossa, Alcian Blue) as well as by RT-PCR analysis on marker genes of differentiation (respectively, C/EBPα, osteocalcin, aggrecan) as previously described [1]. MCs (i.e. Cultispher-S, Cytodex-3, Cytopore-2) were prepared and cells were seeded as reported in [2]. Cell counting was performed as follows: (1) after a full digestion of Cultispher-S by trypsin and using trypan blue exclusion counting method, as in [2]; (2) by crystal violet staining and nuclei counting for Cytodex-3, as in [2]; or (3) cell counting on Cytopore-2 was performed using MTT according to [3]. The multiplication ratio was calculated as defined elsewhere [2]. Cell cycle was analyzed by FACS, after cell staining with propidium iodide, as in [2]. The actin organization was assed by confocal microscopy after cell staining with phalloidin-rhodamine.

## Results

### MSC and microcarrier screening

E-MSCs were compared to the “gold standard” BM-MSCs on the basis of their proliferative properties. E-MSCs bear characteristics of progenitor cells: expression of CD73, Sca-1 and Notch-1, and also *in vitro* differentiation potential into mesodermal cell types such as adipocytes, chondrocytes and osteoblasts (not shown). Thus, these cells are *in vitro* functionally analogous to BM-MSCs. This cell population was further selected on the basis of its high intrinsic proliferation potential in monolayer culture, a clear advantage in the field of MSC bioprocessing (Table 1).

Next, we analyzed these cells’ behavior on various types of MCs. Interestingly, both cell types (E- and BM-MSCs) had similar proliferation profiles under all the conditions tested: Cultispher-S>Cytodex-3>Cytopore-2 (Table 1). This validated that E-MSCs are a valuable model for studying MSCs activities on MCs, given their faster growth and easier handling compared to BM-MSCs (Table 1). In addition, Cultispher-S turned out to be the most efficient MC for MSC expansion (Table 1).

**Table 1:** Multiplication ratios of E-MSCs and BM-MSCs on various culture systems. Multiplication ratios of E-MSCs and percentage of cells in S-phase at day 5 of a 7 day run, under various modes of culture

***MSC and MC screening***			
BM-MSCs	*Culture system*	*Multiplication ratio*	
	
	T-Flasks	0.4 ± 0.2	
	Cultispher-S	0.16 ± 0.1	
	Cytodex-3	-0.1 ± 0.12	
	Cytopore-2	-0.5 ± 0.04	

E-MSCs	T-Flasks	2.4 ± 0.1	
	Cultispher-S	2 ± 0.3	
	Cytodex-3	0.7 ± 0.6	
	Cytopore-2	-0.6 ± 0.3	

***Maximization of MSC proliferation***			

E-MSCs on Cultispher-S	*Mode of culture*	*Multiplication ratio*	*% of cells in S-phase at day 5*
	
	Batch	1.5 ± 0.3	1.4 ± 0.3
	Cyclic-fed-batch	2.6 ± 0.2	8 ± 1
	Pulsed culture	3 ± 0.04	15 ± 1

### Maximization of MSC proliferation in MC-based stirred bioreactors

According to Table 1, a batch culture mode was not sufficient to promote efficient E-MSC propagation on Cultispher-S. Conversely, cyclic fed-batch increased E-MSC growth span (Table 1). In addition, the use of high levels of growth factors (using a pulsed culture composed of 40% FBS and 1 ng/mL of TGFβ1) increased growth span (Table 1). The beneficial effects of cyclic fed-batch and pulsed culture were linked to a sustainment of the percentage of cells in S-phase of the cell cycle compared to batch culture (Table 1). These results underline that the control of growth factor levels in the medium is the key to maximize E-MSC growth extent.

### Sequential proliferation and differentiation in MC-based stirred bioreactors, modulating actin organization

**Figure 1:**
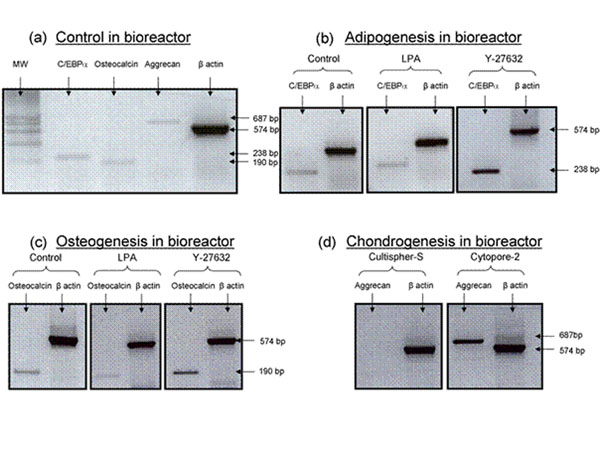
RT-PCR analysis of E-MSC differentiation. Spontaneous marker gene expression after MC expansion (control) (a); adipogenic differentiation (C/EBPα as a marker) after MC expansion, adding an actin modulator in the differentiation medium (Y-27632 or LPA) (b); osteogenic differentiation (osteocalcin as a marker) after MC expansion, adding an actin modulator in the differentiation medium (Y-27632 or LPA); chondrogenic differentiation (aggrecan as a maker) after MC expansion, comparing Cultispher-S to Cytopore 2 (d)

We have previously shown that the addition of differentiation media significantly diminished E-MSC proliferation. This indicated that the differentiation of E-MSCs on MCs must be performed sequentially, after an initial proliferation phase. According to Figure 1.b, after a first step of E-MSC expansion on MCs, it was shown that the repression of fibrillar actin (adding Y-27632 to the differentiation medium) maximized adipogenic differentiation on Cultispher-S, while the promotion of stress fibers (using lysophosphatidic acid, LPA) diminished it. In the same vein, Y-27632 improved E-MSC osteogenic differentiation, while LPA lowered the expression of osteocalcin (Figure 1.c). Cytopore-2, previously shown to promote disorganized actin form (similar to that of aggregate cultures and E-MSCs treated with cytochalasin-D), enabled an efficient E-MSC chondrogenic differentiation, in comparison to Cultispher-S (Figure 1.d). This latter MC was previously found to enable a proper E-MSCs actin organization (composed of mixed cortical and fibrillar actin) linked with their efficient propagation. These results indicate that a tight control of the E-MSC microenvironment leading to adapted actin shape is the key towards efficient MSC differentiation on MCs.

## Conclusions

According to these data, it emerges that a correct control of MSC microenvironment in terms of MC composition is necessary to promote these cells’ efficient proliferation via proper actin organization. An efficient MC system must also be combined with adapted biochemical signaling. Indeed, the growth factor content is an essential factor to monitor towards improved MSC growth yield. As we observed that the differentiation step could not be combined with expansion, sequential phases are required for the mass scale production of a given MSC differentiated phenotype. Similarly to the expansion phase, the microenvironment to which MSCs are exposed modulates the efficiency of their differentiation. According to our results, the promotion of an adequate actin organization is one of the essential parameters enabling, in association to biochemical signaling from the differentiation medium, efficient MSC differentiation on MCs.

Taken together, these results open the way toward mass scale production of MSCs suitable for future *in vivo* applications.
